# Risks Associated with the Presence of Polyvinyl Chloride in the Environment and Methods for Its Disposal and Utilization

**DOI:** 10.3390/ma17010173

**Published:** 2023-12-28

**Authors:** Marcin H. Kudzin, Dominika Piwowarska, Natalia Festinger, Jerzy J. Chruściel

**Affiliations:** 1 Łukasiewicz Research Network—Lodz Institute of Technology, 19/27 Marii Sklodowskiej-Curie Str., 90-570 Łódź, Poland; marcin.kudzin@lit.lukasiewicz.gov.pl (M.H.K.); dominika.piwowarska@edu.uni.lodz.pl (D.P.); natalia.festinger@lit.lukasiewicz.gov.pl (N.F.); 2 Circular Economy Center (BCG), Environmental Protection Engineering Research Group, Brzezińska 5/15, 92-103 Łódź, Poland; 3 Doctoral School of Exact and Natural Sciences, University of Lodz, 21/23 Jana Matejki Str., 90-237 Łódź, Poland; 4 UNESCO Chair on Ecohydrology and Applied Ecology, Faculty of Biology and Environmental Protection, University of Lodz, 12/16 Banacha Str., 90-232 Łódź, Poland; 5 European Regional Centre for Ecohydrology of the Polish Academy of Sciences, 3 Tylna Str., 90-364 Łódź, Poland

**Keywords:** poly(vinyl chloride), PVC, pollution, aquatic environment, soil, degradation

## Abstract

Plastics have recently become an indispensable part of everyone’s daily life due to their versatility, durability, light weight, and low production costs. The increasing production and use of plastics poses great environmental problems due to their incomplete utilization, a very long period of biodegradation, and a negative impact on living organisms. Decomposing plastics lead to the formation of microplastics, which accumulate in the environment and living organisms, becoming part of the food chain. The contamination of soils and water with poly(vinyl chloride) (PVC) seriously threatens ecosystems around the world. Their durability and low weight make microplastic particles easily transported through water or air, ending up in the soil. Thus, the problem of microplastic pollution affects the entire ecosystem. Since microplastics are commonly found in both drinking and bottled water, humans are also exposed to their harmful effects. Because of existing risks associated with the PVC microplastic contamination of the ecosystem, intensive research is underway to develop methods to clean and remove it from the environment. The pollution of the environment with plastic, and especially microplastic, results in the reduction of both water and soil resources used for agricultural and utility purposes. This review provides an overview of PVC’s environmental impact and its disposal options.

## 1. Introduction

Plastics are ubiquitous materials used in a wide range of human activities due to their durability, low cost, and technological versatility [1].

In 2020, about 368 million tons of plastics were produced in the world. Moreover, almost 80% of plastic waste was discharged directly or indirectly into the environment [2,3,4]. Such uncontrolled disposal of materials can cause serious environmental damage, especially to the atmosphere, agricultural soils, and groundwater [5,6].

Environmental factors such as wind, sunlight, and rain can cause the degradation of polymers, leading to the formation of small and durable particles: microplastics (MPs) with a size of 1–1000 μm and nanoplastics (NPs) with a size of 1–1000 nm [7,8]. It is important to detect MPs in the environment as soon as possible to avoid the biological damage they cause. The amount and type of plastics in the environment are assessed, among other things, using Fourier transform infrared spectroscopy, time-of-flight secondary ion mass spectrometry, thermogravimetric analysis technique, differential scanning calorimetry, scanning electron microscope, atomic force microscopy, water contact angle, and ion chromatography [6].

Poly(vinyl chloride) (PVC) is one of the six commonly used plastics (which accounts for as much as 10% of global plastic production) [2]. PVC is a popular plastic due to its low price, durability, and good mechanical, chemical, electrical, and thermal properties. The global production of PVC in 2009 amounted to approximately 34 million tons. At the global level, PVC production in 2015 exceeded 35 million tons, and the annual growth was forecast at approximately 2%. At that time, the European PVC consumption was approximately 7 million tons per year [9]. In 2022, PVC capacity was 59.97 million tons. The market is expected to achieve an annual growth of more than 3% during 2022–2027 [10].

This plastic is strong, durable, long-lasting, lightweight, and versatile, so it is widely used in many industries, such as in construction, automotive industry, pipes and cables, and household goods. The service life of PVC in construction is more than 10 years [11]. PVC can present a number of challenges at various stages of its life cycle, particularly at the waste stage. Sound waste management and disposal are essential due to the potential emission of PVC additives (e.g., heavy metal compounds) into the air (in the case of incineration) and into the soil (in the case of landfilling) but also due to illegal dumping and incineration. Various PVC additives also have hazardous properties and, therefore, when emitted, can pose a threat to the environment and human health [12].

PVC is considered as the most environmentally damaging plastic and one of the most toxic substances for inhabitants of our planet. From cradle to grave, the PVC lifecycle (production, use, and disposal) results in the release of toxic, chlorine-based chemicals, and it is one of the world’s largest dioxin sources. These toxins build up in water, air, and food chains. They cause severe health problems, including cancer, immune system damage, and hormone disruption. Everyone has measurable levels of chlorinated compounds (toxins) in their bodies [13,14,15,16,17].

## 2. PVC Characteristics

**Figure 1:**
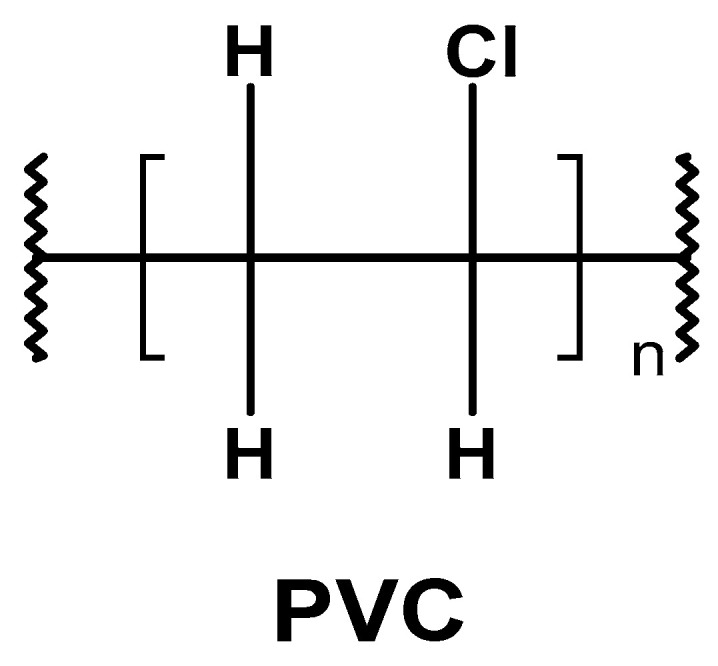
General structure of poly(vinyl chloride) (PVC).

According to the IUPAC, the systematic name of PVC is poly(-1-chloroethylene) (Figure 1) [18,19,20]. It presents the linear, in most atactic structure of polymer chains, with the degree of polymerization ranging from 500 to 1500, corresponding to a theoretical molecular weight range of about 31,000–94,000 g/mol (Da). Poly(vinyl chloride) is white in color and a relatively stiff plastic with a high resistance to impact, chemicals, corrosion, water, and weather conditions [21].

**Figure 2:**
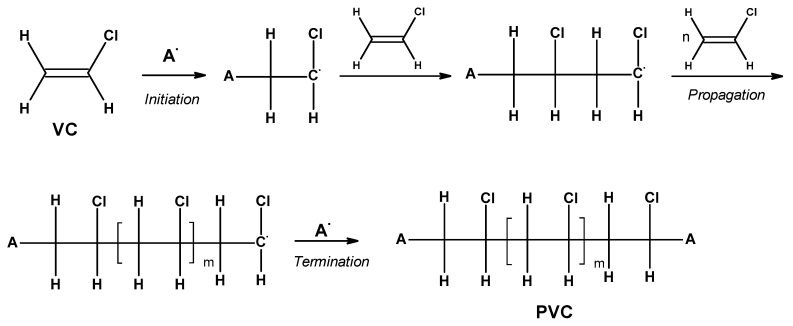
The free-radical polymerization of vinyl chloride (VC) (m = n + 1).

PVC is synthesized through free-radical polymerization occurring via the head-to-tail tri-stage mechanism, depicted in Figure 2 [22].

In industrial practice developed since 1930 [23,24], vinyl chloride (VC) is polymerized in suspension processes (approximately 80% of the market), emulsions (~10–15%), bulk (~10%), and solution (~1%), respectively [20,25]. PVC production uses about 40 percent of the worldwide chlorine production, i.e., ~16 million tons of chlorine per year [26].

PVC has been one of the most widely used plastics for decades. PVC is classified into two broad categories: rigid PVC (unplasticised PVC, uPVC, rPVC, RPVC; used for automobile, health care, electronics, building and construction) and flexible PVC (fPVC; used for cables, wires, fittings, films, profiles, tubes, pipes, sheets, and bottles) [27]. Moreover, chlorinated PVC, molecularly oriented PVC, and modified PVC [28,29,30] are also produced on a smaller scale.

Pure PVC, due to its mechanical properties, requires some additives for the improvement of its processability and application needs. The most common additives used in PCV processing are, in particular, focused on the following chemical products: plasticizers, thermal stabilizers, fillers, impact modifiers, and/or pigments [31].

The list of representative plasticizers used in the PCV industry is given in Table 1.

### 2.1. PVC’s Physical Properties

PVC is classified as a self-extinguishing material. The limiting oxygen index (LOI) of the rigid PVC is approximately 44–49% [34].

The solubility of PVC is one of the major factors facilitating PVC leaching. Thus, the satisfactory resistance of PVC in an aqueous environment is caused by its insolubility in water [35]. Conversely, PVC is not resistant to the majority of organic solvents due to its substantial solubility, which are mostly: tetrahydrofuran (THF), dimethylformamide (DMF), dimethylacetamide (DMA), and/or pyridine [36].

### 2.2. PVC’s Chemical Properties

**Figure 3:**
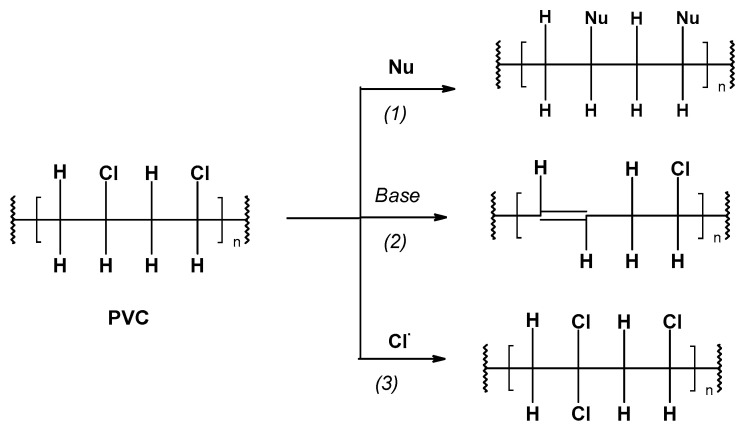
Reaction paths of PVC: the nucleophilic substitution of chloride atom (1), nucleophilic elimination of hydrogen chloride, (2) and free-radical chlorination (3).

PVC, having a polychloroalkane structure, exhibits typical alkyl chloride reactivity, namely, it undergoes the nucleophilic substitution of chloride atom (1) [37,38], nucleophilic elimination of hydrogen chloride (2) [39], and free-radical chlorination (3) [40], which is illustrated in Figure 3. These reactions, due to PVC’s insolubility in water [35], seem to occur on the polymer surface, facilitating its leaching.

**Figure 4:**
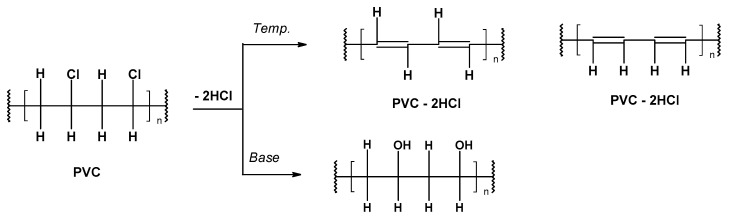
The degradation of PVC by dehydrochlorination reactions.

The degradation of polyvinyl chloride (PVC) is mainly caused by the thermal dehydro-chlorination reaction (Figure 4), leading to the formation of conjugated double bonds or chlorine substitution (hydrolytic degradation) [41,42,43].

PVC waste is resistant to decomposition in the environment due to its high molecular weight, highly stable covalent bonds, and hydrophobic surface properties, creating a huge environmental problem during its production and disposal [6]. PVC waste is highly resistant to decomposition in the environment but often releases harmful chlorinated compounds that negatively affect the health of organisms and the ecosystem [8]. PVC is classified as a substance with strong mutagenic and carcinogenic properties, and it is more toxic than other plastics due to the presence of chlorine atoms in it [44].

**Figure 5:**
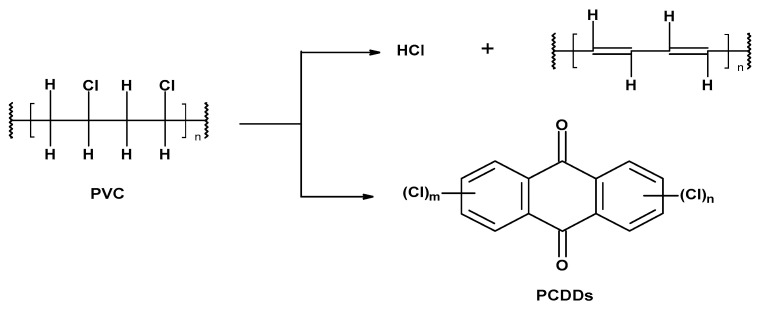
The formation of harmful chlorinated compounds (PCDDs, where *m* and *n* can range from 0 to 4) by the decomposition of PVC.

Traditional methods for the storage and incineration of PVC waste lead to the release of harmful chlorinated compounds: hydrogen chloride and chlorine-containing dioxins (Figure 5) [45,46].

Pure PVC can only be used in the temperature range of −10 to +60 °C. The stabilized softened PVC can be used in the temperature range of −30 to +100 °C. The greatest problems are encountered during the processing of PVC because the thermal decomposition of PVC with the release of hydrogen chloride begins visibly at the temperature of 135 °C. In addition, the released hydrogen chloride in the presence of oxygen from the air accelerates the polymer decomposition process to such an extent that, at the temperature of 200 °C, the PVC decomposes almost completely [47].

In the thermal degradation of vinyl polymers, cleavage outside the main chain or the statistical cleavage of the main chain may occur. Light-induced ageing of PVC occurs as a result of the absorbed light energy, enabling photolysis, oxidation, and cross-linking reactions. PVC-based products used under atmospheric conditions are exposed to the destructive effects of atmospheric factors, such as UV radiation, precipitation, and temperature changes leading to the physical and chemical ageing of the material. Under the influence of UV radiation, free radicals are formed, causing the cleavage of the covalent bonds in PVC’s main chain, the splitting of hydrogen chloride, and the formation of new double bonds, causing the material to turn yellow. The next stage of the degradation is the photo-oxidation process leading to the whitening of the material and cross-linking due to the formation of oxygen bridges. The most characteristic changes caused by the degradation are the appearance of newly formed C=C double bonds, C=O carbonyl groups, and hydroxyl groups –OH in the chain, as well as the release of hydrogen chloride and carbon dioxide. The yellowing of the material is caused by a high temperature and low humidity. The PVC degradation process is a complex phenomenon resulting primarily from the dechlorination process, the course of which depends on the stabilizers used. PVC is the largest contributor to environmental pollution with dioxins on a global scale [47].

There are two types of the degradation of PVC [47]:

- Preliminary (occurring under the influence of temperature, called thermal) under anaerobic conditions at the molecular or ionic level,
- Secondary as a result of the increased temperature and oxygen action (thermo-oxidative degradation).

Because of the many concerns raised about PVC usage since the 1970s, PVC has become one of the most researched plastic materials from an environmental point of view. Despite all the technical and economic problems and the public discussions on the environmental dangers and hazards of chlorine chemistry, poly(vinyl chloride) (PVC) is the second most produced plastic (with a worldwide capacity of about 60 million tons in 2022 [10]), being behind polyolefins and before styrene polymers [10]. But PVC also takes an important part in many environmental discussions on polymers, e.g., chlorine chemistry, toxicity of vinyl chloride, or waste and recycling problems. For a time frame of 70 years, some recent developments in the controlled polymerization of vinyl chloride, stabilization, modification of bulk properties, and chemical and material recycling of PVC are discussed.

### 2.3. Biological Activity of Poly(vinyl chloride)

Plastic products contain hundreds of potentially toxic chemical additives, which currently drive toxicity [48,49]. Micro- and nano-plastics may pose dangers of acute toxicity, (sub)chronic toxicity, carcinogenicity, genotoxicity, and developmental toxicity [50]. This results from the polymer matrix, additives, degradation products, and adsorbed contaminants.

In contrast to its carcinogenic monomer [51,52,53,54], PVC is biologically inert due to its low chemical reactivity [49]. For instance, it is not digestible, even at the surface, for common strains (*Lactobacillus acidophilus; L. plantarum*, and *L. rhamnosus*) representing functional bacterial groups in the human gut microbiota. However, cytotoxicity investigations of PVC using the human cell lines Caco-2, HepG2, and HepaRG (a possible impact on intestine and liver) [55,56,57] and pulmonary cell cultures revealed the induction of cytotoxic effects [6]. Occupational exposures at a poly(vinyl chloride) production facility are associated with significant changes to the plasma metabolome [58]. PVC micro- and nano-particles can induce carcinogenesis to humans [59].

It has been reported that PVC particles produce moderate in vitro toxicity for human pulmonary cells [60,61] and cardiometabolic toxicity [62]. PVC induces changes in the microenvironment and secondary structure of human serum albumin (HAS) (decrease in α-helix) [63] and bovine serum albumin (BSA) [64], and it causes liver injury and gut microbiota dysbiosis [65,66]. PVC dust exerts a hemolytic effect on lung fibroblast cultures [67], and it causes liver angiosarcoma and lung cancer [51].

The toxicity of water after contact with PVC materials was found to be dependent on the type of PVC composition components and temperature [68,69,70,71]. The aquatic toxicity of PVC microplastics towards marine organisms (microalgae, crustaceans, and echinoderms) is due to the leaching of chemical additives and not the ingestion of microplastics (MPs) [72,73]. During the ageing process of MPs, additives are released that may cause severe genotoxicity [74].

PVC microplastics reduce sediment catalase, polyphenol oxidase (PO), and urease activities and decrease physicochemical indicators, including total organic carbon (TOC), total nitrogen (TN), and pH value [75].

Studies on a combustion gas of thermoplastic poly(vinyl chloride) showed its cytotoxicity to human fetal lung tissue cell (MRC-5), African green monkey kidney cell (Vero), and Chinese hamster ovary cell (CHO), with molecular chlorine as the major toxicant [76].

PVC composites for linoleum are equipped with antibacterial agents (e.g., 1,3-dioxanes, wollastonite, or quaternary ammonium biocides); therefore, they exhibit functions of biostatics, inhibiting the growth of microorganisms and bactericides, killing the microorganisms [77,78].

## 3. Threats Related to Production and Use of PVC

Due to its high durability, corrosion resistance, low price, and ease of installation, poly(vinyl chloride) (PVC) finds many practical applications not only in industry but also in everyday life [79]. The largest PVC sales markets include the production of window profiles (27% of the total PVC application in Europe) and pipes, e.g., for drinking water (22% of the total PVC application in Europe). According to the inventory model of PVC production, a total of 2.4638 kg of wastewater is generated during the production of 1 kg of poly(vinyl chloride) from suspension polymerization (S-PVC) (Table 2).

The life cycle of PVC (from the extraction of the raw materials to the end of the production) is considered to be harmful to the environment. This is due to the fact that many pollutants are involved in the entire process [80]. In addition, it is believed that an important phase of the potential environmental impact of PVC pipes is also their installation [82].

It has been shown that the production of PVC has a significant share, e.g., in human toxicity potential (HTP), photochemical ozone creation potential (POCP), acidification potential (AP), and global warming potential (GWP) (Table 3) [83,84].

During the production of 1,2-dichloroethane from ethylene and chlorine, dioxins can be formed in the thermal cracking process of vinyl chloride monomers (VCMs). This is reflected in the HTP values. The higher values recorded for POCP are due to the processing of crude oil, which causes the emissions of volatile organic compounds (VOCs).

The largest amounts of energy needed for the production of PVC are consumed during ethylene production processes. Also energy-intensive is chlorine production, which causes CO_2_ and SO_2_ emissions and contributes to high GWP and AP values [84]. A simulation conducted by Ye et al. showed that chlorine, carbon dioxide, and nitrogen oxides are the key compounds from PVC production contributing to the overall environmental burden [85]. For example, poly(vinyl chloride) in China is mainly produced using coal-based technologies with approximately three times higher CO_2_ emissions than generated in the case of oil-based technologies. In 2017 alone, the Chinese PVC industry emitted an estimated 123.54 million tons of CO_2_, three-quarters of which came from coal-based production [86].

It has also been shown that plasticizers such as phthalates or citrates added to PVC can migrate to solid, gas, or liquid media which come in contact with the plastic. The cause of the migration process may depend on, e.g., the molecular weight of the polymer, the nature and amount of the plasticizer, and environmental conditions [87].

Analyses conducted on the leaching of dibutyl phthalate (DnBP) from PVC microplastics in aqueous solutions corresponding to water and soil environments showed the relationship between the amount of released plasticizer and the size of polyvinyl chloride particles, concentration of added phthalate, as well as ageing of the plastic. The release of phthalates was higher for smaller particles and particles with a higher content of plasticizer. While the ageing of plastics due to solar radiation can either increase the release of phthalates (by increasing plastic hydrophilicity) or decrease it (by reducing readily available fractions), a solution’s pH and ionic strength have little effect on this [88]. The relationship between the size of PVC particles and the amount of additives released from it was also shown by Ye et al. [89].

Smaller PVC fragments release greater amounts of di-n-butyl phthalate than larger fragments. It has also been proven that temperature is a factor that affects the rate and size of migration much more than, for example, exposure to light or the particle size, and with increasing temperature (from 4.25 to 45 °C), both the speed and migration frequency increase [89]. Analyses conducted by Skjevrak et al. [90] showed that from PVC pipes, volatile organic compounds (VOCs) were also released into water. Identified compounds included hexanal, octanal, nonanal, and decanal. Only trace amounts of hexanal and octanal were found in the analyzed water, while nonanal and decanal were present in concentrations ranging from 170 ng/L to 280 ng/L. The volatile compounds, 2,3-dichloro-1-propanol and dichloroacetic acid, have also been detected in bottled water. Moreover, the vinyl chloride monomer, benzene and the semi-volatile organic compounds such as di-n-octyl adipate and bis(2-ethylhexyl) phthalate, were also identified in some samples [91]. The use of intramolecular diffusion (IPD) and aqueous boundary layer diffusion (ABLD) models showed that the diffusion-determining process for continuous phthalate leaching is ABLD, and a high diffusion coefficient of PVC (~8 × 10^−14^ m^2^/s) enhanced IPD. Although the desorption half-lives of the tested PVC microplastics are expected to be longer than 500 years, under the influence of environmental factors, they can undergo strong changes, and the microplastics themselves are a long-term source of phthalates in the environment [92]. The combustion process also has a significant impact on the release of volatile compounds from PVC. The results of pollutant emission tests from burning pipes showed the presence of chlorinated compounds, i.e., hydrogen chloride, chlorine dioxide, methyl chloride, methylene chloride, allyl chloride, vinyl chloride, ethyl chloride, 1-chlorobutane, tetrachlorethylene, or chlorobenzene. The analysis of leachate from PVC pipes showed the presence of 40–60 components, mainly belonging to long-chain hydrocarbons (e.g., tetradecane, hexadecane, octadecane, docosane) [93].

The long life and a high durability of PVC compared to other plastics make it one of the most common plastic wastes in the environment. The estimated durability of PVC pipes is over 100 years [82]. Nevertheless, the large amount of the additives used in the production of PVC, its susceptibility to weathering processes, and the use of various advanced treatments required for its isolation from environmental samples lead to changes in the surface of PVC microplastics, which may result in large underestimations of its amounts in environmental studies [94].

## 4. Worldwide Pollution of the Aquatic Environment by the Poly(vinyl chloride) Industry

Their durability and low weight mean that plastic microparticles are easily transported along with air and water currents. A research on a floating debris in the ocean clearly showed that plastics are the most common type of pollution found in surface waters. Numerous methods for measuring microplastics in aquatic environments have been published recently [95,96,97]. Thus, Pan’s report on microplastics present in the Northwestern Pacific indicated the ubiquity of MPs with an average abundance of 1.0 × 10^4^ items km^−2^. A micro-Raman spectroscopic analysis of the MP samples collected indicated that the dominant MPs were polyethylene (57.8%), polypropylene (36.0%), nylon (3.4%), polyvinyl chloride (1.1%), polystyrene (0.6%), rubber (0.9%), and polyethylene terephthalate (0.2%) [98].

Due to the fact that PVC is one of the most commonly used synthetic polymers and is characterized by a high density, not only does it occur in surface waters, but it is also particularly identified in sediments [97,99,100]. Representative papers on environmental pollution by PVC are presented in Table 4.

## 5. Toxicity of Poly(vinyl chloride) to Surface Water Trophic Networks and Humans

Most of the additives are not covalently bound to the plastic; therefore, they can easily migrate to the surface of the material and be released into the environment [136,137]. The additives exhibiting toxic properties towards organisms can be distinguished as bisphenols [138] and phthalates [139], which are indicated as potential endocrine-disrupting chemicals (EDCs) in both animals and humans [140]. The monomer used in the production of PVC—vinyl chloride (VCM) is also highly toxic and cancerogenic [141]. VCM is released into the workplace and into different environments and causes cancer in laboratory animals [26].

During the PVC lifecycle, very large quantities of hazardous organochlorine by-products are formed accidentally and released into the environment. They are highly persistent, bioaccumulative, and toxic, and they have been shown to cause a range of health hazards, which, in some cases, are at extremely low doses, including the following:

- Cancer,
- Disruption of the endocrine system,
- Reproductive impairment,
- Impaired child development and birth defects,
- Neurotoxicity (damage to the brain or its function),
- Immune system suppression.

Most important by-products of the PVC lifecycle are dioxin (2,3,7,8-tetrachloro-dibenzo-p-dioxin) and a large group of structurally and toxicologically related compounds, generally called dioxins or dioxin-like compounds. Dioxins are global pollutants found in the tissues of whales in the deep oceans and polar bears in the high Arctic. Human infants receive particularly high doses (orders of magnitude greater than those of the average adult) because dioxins cross the placenta easily and concentrate in breast milk [26].

Phtalate plasticizers used in PVC technology leach out of PVC-based materials. They can be found in the water of the deep oceans, air in remote regions, and the tissues and fluids of the general human population. Phthalates can damage the reproductive system, causing infertility, testicular damage, reduced sperm count, suppressed ovulation, and abnormal development and function of the testes and male reproductive tract in laboratory animals. They cause cancer in laboratory animals. The phthalates used in processing PVC may lead to asthma. Metal stabilizers present in PVC are also highly toxic and are not degraded in the environment [26].

The effect of humic acid (HA) on the photo-ageing process of poly(vinyl chloride) microparticles (PVC-MPs) was studied using FTIR spectroscopy. The ecological risk was assessed through bioassays. The presence of HA increased the rate of the photo-ageing of PVC-MPs. Moreover, the chemicals leaching from PVC-MPs showed higher acute and genetic toxicities under light irradiation in comparison to dark conditions. The presence of HA significantly enhanced the biotoxicity of the leachate, indicating the ecological risk of MPs in surface waters [142].

It was estimated that rivers are responsible for the transport of 70–80% of the total amount of plastic that ends up in oceans, where they interact with see animals. The data from 778 publications based on field and experimental studies (LITTERBASE) were summarized by Tekman et al. [143].

Most of it comes from manufacturing processes, agriculture, and wastewater treatment plants discharging wastewater into water systems [144]. Microplastics, such as PVC, once released into the environment, pose a serious threat to ecosystems [145,146]. Due to their small size (<5 mm), the microplastics are easily absorbed by biota, causing negative effects on their development, reproduction, and survival [147,148,149]. It has been shown, among others, that PVC MPs accumulate and negatively affect such aquatic organisms as invertebrates [99], zooplankton [150], or fish [104,151,152] (Table 4).

The harmful effects that microplastics can cause on aquatic species include neurotoxicity, behavioral changes, histopathological damage, biochemical and hematological changes, and embryotoxicity (Tekman, 2022) [143]. In addition, due to their size similar to plankton, poly(vinyl chloride) and other microplastics are easily absorbed by aquatic organisms from various trophic levels, and they are accumulated at higher levels, entering the food chain and, thus, posing a threat not only to animals but also to humans [144,153]. Moreover, plastic waste can cause injury and abrasion to coral tissues, thus facilitating the invasion of pathogens such as *Rhodobacterales*, which dominate the biofilm that forms on PVC in the marine environment [143,154].

Plastics not only threaten the survival of more than 800 species of animals, including large marine mammals (e.g., whales and dolphins) and various birds, but also, through the transfer and release of toxic chemicals such as harmful additives, persistent organic pollutants, and heavy metals (e.g., lead), result in the chemical pollution of the environment, invasion of exotic species, and a degradation of local tourism and fishing industries [50,155,156,157,158]. The pathways of plastic debris and microplastic transportation in the environment and their biological interactions were described by Meem et al. [159].

Using the example of *Daphnia magna*, it has been proven that PVC has a negative effect on the reproductive processes of zooplankton. PVC microparticles at concentrations of 10–500 mg/L led to a decrease in reproduction efficiency, and at a concentration of 500 mg/L, it delayed it. The toxic nature of PVC was due to the substances added to it, as it was the chemicals extracted from it that were harmful to the model organism [150]. The delay in the hatching period combined with the occurrence of oxidative stress and changes in the expression of genes related to reproduction and detoxification in *D. magna* exposed to PVC microplastics (PVC-MPs) at sizes of 2 and 50 µm were also demonstrated [160]. Chronic exposure to the tested microplastic, e.g., extended the days to the first brood, increased the total number of broods per female, reduced the number of her offspring, disrupted the activity of superoxide dismutase (SOD) and catalase (CAT), and increased the level of glutathione (GSH). Studies conducted on *Phaeodactylum tricornutum, Chaetoceros gracilis*, and *Thalassiosira* sp. also showed toxic properties of PVC MPs towards representatives of diatoms. The high concentration of PVC (200 mg/L) reduced the content of chlorophyll in the cells and negatively affected the processes of photosynthesis (Table 5). In addition, the PVC MPs caused a physical damage to the diatom’s cell structure [161]. The acute toxicity of PVC to cultured human cell lines was also detected [162].

Since microplastics such as PVC are commonly found in drinking water [174,175,176] and bottled water [177,178], the human body is also exposed to their harmful properties [179,180].

It was estimated that people who consume the recommended daily amount of water only from bottled sources may consume up to 90,000 microparticles of plastics per year, compared to 4000 microparticles of plastics consumed by people drinking only tap water [181]. Sources of human exposure to microplastics and their impact on aquatic organisms were described by Gola et al. [182]. In addition to the exposure to MPs along with the water contaminated with them, the ways in which humans are exposed to MPs include the consumption of seafood, the global per capita consumption of which is over 20 kg per year [183]. For example, research results have indicated that the exposure of an average Irishman to the microplastics accumulated in seafood intended for consumption ranges from 15 to 4471 particles per year [131]. Other estimates indicate that the maximum amount of microplastic particles absorbed by humans from seafood is 53,864 particles per year. These data were based on global consumption estimates of 15.21 kg of fish per year/person, 2.65 kg of molluscs per year/person, and 2.06 kg of shellfish per year/person [184].

Microplastics are also common in commercially available sea salt obtained from the Atlantic Ocean and Mediterranean Sea, and the most frequently identified molecules in the study by Fischer et al. were polypropylene and poly(ethylene terephthalate) (in 16 out of 17 samples), polyethylene (15/17), polystyrene (13/17), and poly(vinyl chloride) (7/17) [185]. The potential human exposure to the microplastic particles found in sea salt was estimated to be 0–1.674 particles/year [186].

## 6. Contamination of Soil with PVC (and Other Plastics)

Agricultural soils are deeply affected by pollution from microplastics and synthetic polymers. Contaminants arise from bead fragments, plastic mulches, films, fibers, or biodegradable plastics [187]. Plastics are used in agriculture in order to improve production and ensure food safety for human health. The microparticles of polyethylene (PE), polyamide (PA), polystyrene (PS), polypropylene (PP), polyethylene terephthalate (PET), and poly(vinyl chloride) (PVC) are commonly found in the soil [188,189]. The microplastics are subject to accumulation, which can be reduced by using biodegradable materials [190]. Their accumulation in the soil is much higher than their accumulation in aquatic habitats. It is estimated that the amounts of plastic in the soil worldwide is equal to about 0.6 mgkg^−1^ of dry soil [6]. Sources of the plastic contamination of the soil may include illegal dumping, irrigation with contaminated water, and atmospheric deposition [8,191].

The plastics can release toxic chemicals into the soil, which then seep into ground water [192]. They reduce water percolation, limit the aeration of the soil, and contribute to climate change by releasing large amounts of CO_2_ into the atmosphere during their production and degradation. The effect of plastics on the physicochemical properties of the soil depends on their type, amount, shape, and particle size [8]. Since a polymer’s surface is sometimes charged, plastics can interact with charged ions found in the soil [193]. The large amounts of the microplastics present in the soil negatively affect the biophysical and chemical properties of the soil, including the structure, porosity, texture, pH, surface area, and nutrient content [194]. Polymer particles can affect soil aggregation, which leads to the loss of the soil strength. Due to the lack of oxygen below the soil surface, the polymer aggregation is characterized by the slow rate of biodegradation. Importantly, the biodegradation process under anaerobic conditions produces methane and carbon dioxide [195]. The MPs adsorb or release heavy metals during ageing. Any changes in the soil pH and heavy metal availability due to microplastic particle contamination may be indirectly related to soil microbial activity [196]. The presence of PVC in the soil significantly increases the rate of soil nitrification and nitrate reductase activity, which may further promote soil denitrification [197]. Recent studies have indicated that PVC increased the NH_4_^+^-N content and decreases the NO_3_-N content [198]. The relative abundance of ammonium-oxidizing and denitrifying bacterial groups changed significantly after the addition of MPs. Moreover, the presence of PVC significantly increased the richness of denitrifying bacteria in the soil [188]. Microplastics react with natural fertilizers such as cow manure, causing a greater impact on natural greenhouse gas emissions such as nitrous oxide, ammonia, carbon dioxide, and methane [199].

Due to limited photooxidation and limited oxygen conditions necessary for degradation, microplastics can persist in the soil for more than 100 years [8]. MPs are also toxic when ingested by living organisms. A major danger is their accumulation in a biological chain [74]. Studies have indicated that MPs smaller than 130 µm accumulate in human tissues and release toxic substances, affecting the function of organs such as the liver and lungs [180].

Different types of microplastics can exhibit different behaviors, causing different impacts on soil ecosystems [200]. Microplastic particles with a size of 50 nm taken up by plants can penetrate the roots, where the spherical plastic particles with the size of about 2 μm and a low degree of mechanical flexibility have been identified [190].

It was found that PVC caused a significant reduction in the fresh weight of shoots and a decrease in the number of fruits of the plant *Solanum lycopersicum L*.; it caused a marked increase in Ni and Cd contents, as well as the decrease in nutritionally valuable lycopene [201]. PVC is more dangerous than other MPs—it affected the nutritional properties of the *Capsicum annuum* plant, reduced the maximum protein content, and strongly affected contents of vitamin A and vitamin B6 in the fruit. Moreover, PVC reduced the content of oleic and linoleic acids and seriously degraded the total content of flavonoids and phenols. The effect of PVC on the macro- and micro-nutrients of the *C. annuum* fruit, especially Ca, K, Mg, and Zn, is also not insignificant [202].

Moreover, plastics in the soil can affect the community structure of soil bacteria and fungi, which are important components of soil microbiota [8,147]. Thus, they play a fundamental role in regulating soil ecosystem functions. Soil bacteria and fungi have different sensitivities to environmental disturbances [8]. PVC reduces the diversity of microflora in the soil rhizosphere [200]. Earthworms are a key factor in maintaining a healthy soil ecosystem. These invertebrates modify the hydraulic properties of the soil and lead to microplastic transport through the formation of burrows, as has been demonstrated for the earthworm *Lumbricus terrestris* [203]. Microplastic particles affect a wide range of organisms, such as protists, flagellates, ciliates, nematodes, ameba, and isopods [204], and they can restrict their movement by attaching to their external body surface. Their ingestion can cause gastrointestinal damage, reduced responses, poor metabolism, reduced fitness and growth, and increased fatality in the earthworms. Exposure to as little as 1% and 2% (wt./wt.) has been shown to have lethal effects [190]. Nematodes were also used to study the effects of microplastics on soil organisms. Contact with microplastic particles on *Caenorhabditis elegans* caused the inhibition of the body length and reproduction rate. They cause permanent intestinal damage due to the reduced calcium levels and an oxidative stress in the gut, which result from the accumulation of the enzyme glutathione S-transferase [205]. Even a one-day exposure of the nematodes to higher concentrations of microplastics significantly affected the number of offspring [190]. Microplastic exposure in the soil was responsible for the reduction of the body weight and reproductive capacity of collembolans and the modulation of Isopods’ immune processes [190,206].

## 7. Disposal Methods of Poly(vinyl chloride) from the Environment

In the face of the increasing levels of environmental pollution from waste plastics, the development of highly efficient and environmentally friendly methods for their degradation is urgently needed. In 2000, the European Commission published a Green Paper for PVC waste [207], which assessed various environmental and health aspects and the possibility of reducing its impact on the environment. It paid particular attention to measures leading to solutions to PVC waste management problems. For example, in the Vinyl2010 Voluntary Commitment, it was suggested to reduce organochlorine emissions through the sustainable use of additives and various controlled-cycle management strategies. Its successor, VinylPlus, set an annual recycling target of 900,000 tons by 2025 and at least 1,000,000 tons by 2030 [11,208].

It seems promising to carry out complete dechlorination processes before degradation. Owing to full dechlorination, PVC can be treated in the same way as common halogen-free plastics [209]. Such methods include chemical modifications, the near-critical methanol process for PVC dechlorination and recovery of additives, and the near-critical process using an aqueous ammonia solution. Among these techniques, the well-known method is the chemical modification of PVC through the substitution of some chlorine atoms with various nucleophilic reagents. Another technique used to convert waste into energy with simple, fast reactions is hydrothermal treatment. In this technique, super- or sub-critical water is used as a solvent and reagent for the reaction of organic compounds [210].

Various additives and solvents such as NaOH or dimethylsulfoxide (DMSO) were used to improve the dechlorination efficiency. However, when large amounts were applied, their recycling caused problems (Table 6) [211].

Moreover, despite the frequent use of certain chemicals in the hydrothermal dechlorination of PVC waste, their role in this process has not been fully understood. Analyses conducted by Zhao et al. with the use of Na_2_CO_3_, KOH, NaOH, NH_3_·H_2_O, CaO, and NaHCO_3_ in water containing Ni^2+^ showed that the alkalinity of the additives has a significant impact on the effectiveness of the dechlorination process. The most effective additive in these studies was Na_2_CO_3_ (concentration 0.025 M), with a maximum efficiency of 65.12% [218]. The processes carried out using subcritical water-NaOH (CW-NaOH) and subcritical water-C_2_H_5_OH (CW- C_2_H_5_OH) proved that the main mechanism in the case of the dechlorination in CW-NaOH is the nucleophilic substitution of hydroxyl group in PVC, while in CW-C_2_H_5_OH—the nucleophilic substitution and direct dehydrochlorination were the equally significant processes [191]. The key parameter of the dechlorination process is temperature. As the efficiency of this process also decreases with a decrease in temperature, the above-mentioned additives were used to improve the efficiency. Unfortunately, the incorporation of the additives not only increased the costs of the dechlorination process, but also generated secondary pollution [219]. Temperature was also shown to be important in the removal of chlorine (Cl) from PVC in gas–liquid fluidized bed reactor studies where hot N_2_ was used as the fluidizing gas to fluidize the polymer melt [220].

Although poly(vinyl chloride) is a commercially important polymer, it is also one of the most sensitive to UV radiation. A study by Yang et al. showed that the rate of the photoaging of plastics is faster than other ageing processes; therefore, it is one of the most common methods of PVC degradation [221]. The UVA radiation in deionized water, sea sand, and air was used to photodegrade plastics. The results showed that PVC effectively absorbs the UVA radiation in air, and this is where the ageing efficiency was the greatest. The ageing process included photoinitiation, chemical bond breaking, and oxygen oxidation [74,221].

Under the influence of UV radiation (in the wavelength range of 253–310 nm) and in the presence of oxygen and moisture, PVC underwent very rapid processes of dehydro-chlorination and peroxidation to form polyenes. The irradiated material crumbled, lost its stretch, elasticity, and impact resistance, and the surface of the degraded polymer was significantly modified, i.e., loss of abrasion resistance, gloss, and interfacial free energy were observed [212,222].

The use of the photodegradation process makes it easier to dispose plastics from the environment. In order to accelerate the photodegradation of plastics, semiconductor photocatalysts such as TiO_2_, ZnO, Fe_2_O_3_, CdS, and ZnS were also used. For example, it was observed that the addition of ZnO to PVC increased the decomposition of the composite by 4.13% in the case of artificial UV radiation and by 9.7% in the case of solar radiation, respectively [223]. A photodegradable composite film was prepared by doping poly(vinyl chloride) plastic with nano-graphite (Nano-G) and a TiO_2_ photocatalyst. After exposure to the UV radiation (for 30 h), the weight loss rates of Nano-G/PVC, TiO_2_/PVC, and Nano-G/TiO_2_/PVC films were 7.68%, 8.94%, and 17.24%, respectively, while pure PVC decreased its weight by only 2.12% [224].

PVC is less biodegradable than other plastics [225,226]. Therefore, there have been many studies on the thermal decomposition and photodegradation of PVC, but there are a few reports in the literature on the biodegradation of poly(vinyl chloride) compared to other polymers [227], and microorganisms capable of decomposing it, both in the aquatic environment [228] and in the soil, are sought [229].

### 7.1. Recycling and Utilization of PVC

PVC can be recycled using various material and energy recovery methods [230].

Recycling techniques include:

mechanical methods—consisting in extruding and mixing the material with primary polymers,

chemical methods—changing the polymer structure of the material using chemical and thermal agents [231].

The mechanical recycling is the most-recommended way to recycle PVC [43]. The conventional mechanical recycling processes are based on the separation, shredding, and application of shredded material with an unchanged chemical composition to a processing equipment. In this technique, plastics are collected and sorted by hand and/or machines at recycling plants and then flaked in a high-speed mill and cleaned with a detergent and water. Finally, the dry flakes are melted and cast into pellets from which new products can be made [213]. The limitation of the mechanical or secondary recycling is that it cannot be used in the case of the unmodified PVC waste of a known composition and origin [230].

For economic and environmental reasons, the feedstock recycling of PVC is used, including waste that cannot be mechanically recycled. This relatively simple method of PVC recycling allows for energy recovery, which consists of the gasification of fuels or direct combustion in specialized thermal utilization plants. In the case of energy recovery, a fraction of PVC is mixed with other types of waste. The thermal process consists of two steps: dechlorination and the use of the remaining hydrocarbons. Through the thermal recycling of PVC waste, hydrogen chloride is recovered, and other recovered chemicals can find various applications, especially in the chlorine industry [43]. Poly(vinyl chloride) (PVC) waste with a high chlorine content is the source of chlorine, providing hazardous chlorinated organic pollutants, which can be reused as chemicals, fuels, and feedstock [232].

Some new mechanical recycling technologies are based on selective dissolution for the recycling of PVC in an economically feasible way. However, currently, only a small amount of PVC post-consumer waste is being recycled. Incineration, in conjunction with municipal waste disposal, is a simple option that allows for the partial recovery of energy and chemical substances when state-of-the-art technology is applied [233].

One of the common chemical recycling techniques is pyrolysis, divided into hydrocracking, thermal cracking, and catalytic cracking [21]. Although pyrolysis is an effective method for converting PVC waste into energy, it yields products containing significant amounts of chlorine [234,235]. The release of harmful substances such as polychlorinated dibenzo-p-dioxins (dioxins) and polychlorinated dibenzofurans (furans) also occurs in processes such as incineration [231]. During the thermal degradation of PVC, HCl is eliminated, leading to the formation of conjugated double bonds, and it, in turn, attacks other compounds with double bonds, leading to the production of organochlorine compounds [236]. Even if PVC is landfilled instead of incinerated, during the process, it may release, among others, phthalates and heavy metals such as lead, cadmium, and tin [209]. Therefore, these processes, including storage, pose a significant risk of releasing chlorinated organic compounds, microplastics, and pollutants into soils and waters [237]. Due to the low efficiency of the recycling and the tendency to cause secondary pollution, the traditional methods of disposal of the plastic waste—incineration and landfilling—have been banned [231]. Therefore, it has become important to develop techniques to reduce Cl migration.

### 7.2. Biodegradation of PVC Waste

The biodegradation of plastics found in the soil is a complex process. The efficiency of this process is influenced by the availability of substrates assimilable by microbial consortia, molecular weight, surface and morphological characteristics, as well as the structure of the polymers [238]. The biodegradation includes the formation of microbial biofilms on plastic surfaces, followed by the enzymatic degradation of the polymer structure, which leads to the release of oligomers and monomers [8]. The biochemical transformation of resistant polymers by microorganisms usually involves the transformation of complex compounds into simpler forms, leading to a reduction in the molecular weight, as well as the loss of the mechanical strength and surface properties of plastics. The biochemical degradation processes of PVC consists of five stages: colonization, biodeterioration, biofragmentation, assimilation, and mineralization. The first stage of the biodegradation mechanism is the colonization of the microorganisms on the plastic surface. It involves the adhesion of living microorganisms (bacteria and fungi) to the surface of plastics and their use for microbial growth and reproduction. During colonization, the microorganisms form biofilms, which causes damage to the polymer surface [239]. The physical and chemical actions of the microorganisms lead to the biodeterioration and superficial degradation of many kinds of polymers, including PVC. They causes changes in their physical, mechanical, and chemical properties [240].

The prolonged exposure to light, high temperatures, and chemicals in the atmosphere facilitates the biodeterioration process. The microorganisms penetrate the polymers and increase pores and cracks. On the other hand, some microbial species with chemolithotrophic potential promote oxidation and reduction reactions, and chemical biodeterioration [239]. Biofragmentation is a lytic process that allows for the breakdown of polymers into monomers, dimers, or oligomers. The process involves a decrease in the molecular weight of polymers and the oxidation of the lower-weight molecules using specific enzymes (oxidoreductases and hydrolases), as well as free radicals [217]. An enzymatic depolymerization of plastics released monomers that were transported into cells, where they underwent a series of enzymatic reactions leading to complete degradation and the formation of CH_4_, CO_2_, H_2_O, and N_2_ [241]. The mineralization stage can be aerobic or anaerobic, and it was catalyzed by several enzymes: cutinase, laccase, esterase, peroxidase and lipase in a study [239]. PVC-MPs significantly inhibited the chemical oxygen demand (COD) removal efficiency of anaerobic granular sludge (AGS) by 13.2−35.5%, accompanied by 11.0−32.3% decreased formation of methane and 40.3−272.7% increased accumulation of short-chain fatty acids [242].

Examples of the biodegradation of PVC with different kinds of microrganisms [228,243], bacteria (*Pseudomonas, Mycobacterium*, *Bacillus*, and *Acinetobacter*), and fungi (*Basidiomycotina*, *Deuteromycota*, *Ascomycota*) were reported recently. Surface damage and a molecular-weight decrease were observed [214,244,245,246]. Also, an extracellular lignin peroxidase of the fungus *Phanerochaete chrysosporium* showed PVC-degrading activity [247]. Alternatively, the biodegradation of PVC occurred with bacteria isolated from the larva’s gut microbiota (*Spodoptera frugiperda*). Enzymatic assays (e.g., catalase-peroxidase, dehalogenases, enolase, aldehyde dehydrogenase, and oxygenase) caused the depolymerization of PVC [248].

Enzyme specificity and temperature are of great importance in the degradation of plastics. Moreover, the use of several microbial consortia and several enzyme complexes allows for an increase in the biodegradation efficiency compared to a single enzyme or single microorganisms [8]. It is known, however, that the biodegradation of PVC involves three main reactions, including the chain depolymerization, oxidation processes, and the mineralization of the resulting intermediates [249]. An effective approach to the bioremediation of the environment from plastics is their initial thermal treatment. After the thermo-oxidative modifications of PVC, it was noticed that *Achromobacter denitrificans* bacteria isolated from compost were able to eliminate 12.3% of the plastic, which was evidenced by its weight reduction [250].

Of all higher organisms, only some insects are capable of degrading various plastics and converting them into monomeric compounds. In particular, insects in their larval stages have shown the ability to degrade plastics [216]. Insects that metabolize plastic include yellow mealworms (*Tenebrio molitor*), giant mealworms (*Zophobas atratus*), and superworms (*Z. atratus*). It is thought that this unique “plastic-eating” phenomenon may be related to the ability of some of these insects to degrade lignin [251]. The insects have also been shown to ingest polymers, with the actual ingestion being led by the microorganisms inhabiting their guts [239,251,252,253].

Some bacteria, e.g., *Pseudomonas citronellolis*, were capable of degrading PVC films. A 45-day incubation resulted in a fragmentation of the material and a decrease in its average molecular weight by 10%. The maximum weight loss during the further stages of the experiment was 19% [215]. Almost 12% weight loss of PVC was also observed in an experiment conducted in anaerobic microcosms using enriched anaerobic consortia from marine samples (waste and water). In addition, this material showed lower thermal stability after 7 months of incubation [228].

Changes in the mechanical properties of the material were also observed in the analyses carried out using isolates of marine bacteria of the genus *Vibrio, Altermonas* and *Cobetia*. The most effective microorganisms in the elimination of PVC turned out to be the *Altermonas* BP-4.3 strain, in which case, after 60 days of incubation, a 1.76% loss in the weight of the poly(vinyl chloride) film was observed [254]. *Micrococcus luteus* from areas heavily polluted with plastics was able to mineralize 8.87% of PVC. This level was achieved in cultures maintained for 70 days with mineral substrate [255].

The importance of microorganisms in processes such as the depolymerization of PVC was also demonstrated using the example of microorganisms living in the intestines of *Tenebrio molitor* larvae. For biodegradation tests, rigid PVC microplastic powders (MPs) were used (70–150 μm), with weight-, number-, and size-average molecular weights (M_w_, M_n_, and M_z_) of 143,800, 82,200, and 244,900 g/mol, respectively, as the sole diet at 25 °C. The ingested PVC was broadly depolymerized, and the M_w_, M_n_, and M_z_ values decreased by 33.4%, 32.8%, and 36.4%, respectively. After 5 weeks of experiments involving the incorporation of poly(vinyl chloride) into the larvae diet, their survival rate with PVC as the only component of the diet was maintained at the level of up to 80% [243]. The ability of *T. molitor* to eliminate PVC was also confirmed by Bożek et al. after 21 days of the exposure of mealworm to poly(vinyl chloride) in the diet; a 3% loss in the mass of the material was observed [252]. Two bacterial strains isolated from oil-contaminated soil (*Pseudomonas aeruginosa* and *Achromobacter* sp.) showed the ability to degrade PVC containing epoxidized vegetable oil (75% by weight), resulting in a change in the material’s surface topography and a decrease in its tensile strength during an incubation period of 180 days [245]. Some microorganisms are capable of degrading PVC. However, the PVC materials used in these study were largely plastics containing plasticizers. It was found that some bacterial strains acted mainly on the PVC additives, and there was a low ability to degrade PVC without the additives [215].

Apart from bacteria, microscopic filamentous fungi were also tested as organisms potentially capable of degrading PVC. Analyses carried out using *Chaetomium globosum* (ATCC 16021) have shown, for example, that this fungus was able to adhere to the surface of PVC, which was the first stage of the degradation process [214]. An exposure of PVC fragments containing plasticizers (dioctyl phthalate and dioctyl adipate) to the atmosphere for a period of 2 years showed that between the 25th and 40th weeks, the surface of the plastic was dominated by *Aureobasidium pullulans.* After 80 weeks, the next microorganisms identified were, e.g., *Rhodotorula aurantiaca* and *Kluyveromyces* spp. All tested strains of *A. pullulans* grew in the presence of PVC, using it as a carbon source, degrading plasticizers, and producing an extracellular esterase and reducing the substrate weight during growth [256].

Biodegradation potential, through adhesion to poly(vinyl chloride) by *Lentinus tigrinus* PV2, *Aspergillus niger* PV3, and *Aspergillus sydowii* PV4, was also confirmed (Ali, 2014) [257]. For fungi of the genus *Aspergillus* (*A. Niger* Sf1 and *A. glaucus* Sf2), it was observed, among others, that there was 10% and 32% weight loss of PVC over 4 weeks of the experiment, respectively, while for *Bacillus licheniformis* Sb1 and *Achromobacter xylosoxidans* Sb2, with the same observation time, the values were 15% and 17%, respectively [258]. *Phanerochaete chrysosporium* PV1 strain showed the potential for PVC film degradation, for which Fourier transform infrared spectroscopy and nuclear magnetic resonance analysis showed significant structural changes in the material. This was confirmed by peaks corresponding to alkenes appearing, decreases in peak intensity appearing in the case of C–H stretching, and a decrease in the weight of the analyzed PVC itself [257]. The decrease in the PVC weight was also demonstrated during the experiment conducted for 12 weeks with strains isolated from the soil. The loss of 0.064 g/m^2^ for *Mucor hiemalis*, 0.300 g/m^2^ for *Aspergillus versicolor*, 0.341 g/m^2^ for *Aspergillus niger*, 0.619 g/m^2^ for *Aspergillus flavus*, 0.082 g/m^2^ for *Penicillium* sp., 0.240 g/ m^2^ for *Chaetomium globosum*, 0.330 g/m^2^ for *Fusarium oxysporum*, 0.240 g/m^2^ for *Fusarium solani*, 0.364 g/m^2^ for *Phoma* sp., and 0.145 g/m^2^ for *Chrysonilia sitophila* was observed, respectively. The ability of *Mucor* sp. fungi to grow in the presence of poly(vinyl chloride) as the only source of carbon and energy was also demonstrated [259].

With regard to the fact that additives added to polymers may increase their physical and chemical degradation, it has also been shown that the addition of a small amount of cellulose to PVC may cause changes in its properties and facilitate its microbiological degradation [225,260,261].

Under aerobic conditions, vinyl chloride (VC) served as the sole source of carbon and energy for *Pseudomonas putida* strain AJ and *Ochrobactrum* strain TD, which were isolated from hazardous waste sites. Analyses conducted on the biodegradation of vinyl chloride, used as a monomer for PVC production, showed that alkene monooxygenase is responsible for its metabolism in AJ strains of *Pseudomonas putida* and AD *Ochrobactrum bacteria* [262]. The degradation of acetate-modified PVC (PVA) involved, among others, enzymes such as oxidases [263]. The activity of PVA oxidase was correlated with PVA dehydrogenase. The β-diketone group was introduced into the PVA polymer molecule through the product of the reaction carried out by the dehydrogenase. This product, through an active site of serine hydrolase, initiated the oxidation reaction by PVA oxidase. This was followed by hydrolysis to form the monomer [264]. While there is a lot of data on the enzymes involved in the degradation of modified poly(vinyl chloride), scientific reports on the mechanisms and enzymes involved in the degradation of PVC are virtually nonexistent [263]. This is due to the high chemical stability and hydrophobicity of the C-C skeleton of PVC [265]. Among the few data available, there is a mention that, in the case of genus *Cochliobolus*, in the degradation of low molecular weight PVC, laccase is involved [266].

## 8. Conclusions

Polymers and plastics have become an important daily commodity around the world. Demand for them is very high in all industrial sectors. Most of the polymers are insoluble in water and pose a serious threat to the environment because they deplete natural resources and limit their use. Problems of the microplastic pollution of agricultural soils are becoming continuous irrigation with wastewater, the use of sewage sludge, and mulching with plastic films.

Water and soils contaminated with PVC and other plastics pose a threat to living organisms. The microplastics contaminate groundwater and agricultural soils, which is particularly dangerous. They accumulate in the living organisms, thus becoming a part of the food chain.

Due to the slow rate of their biodegradation, all plastics can survive in the environment for centuries. It follows that microplastic pollution is a long-term problem. Methods of the removal of PVC from the environment; its thermal, photo-, and bio-degradation processes; as well as recycling and utilization methods have been described in this article in more detail and a more comprehensive manner than in other reviews.

To the best of our knowledge, the impact of recycling methods on the molecular structure of PVC and the effect of its molecular weight on the chemical structure of the PVC recovered during the recycling processes have not been studied so far. It also seems quite obvious that particles size, molecular weight, and the kind and contents of plasticizers, antiaging agents, and other additives affect the rates of the thermal destruction, photo-, and bio-degradation of PVC, depending on environmental conditions.

## 9. Future Directions

One of the most common commodity plastics in the environment is PVC, which massively contaminates resources. There is no information on the consumption of plastics, including PVC, by livestock. Microplastics can cause adverse effects on animal health and accumulate in their bodies, thus becoming part of the food chain. Special attention should be paid to the aspect of the effects of microplastic pollution on the lives of higher organisms. The widespread pollution of the environment (water, air, and soil) indirectly causes the reduction of natural resources such as water and soil. Polluted water and soils pose a threat to the life and health of all living organisms, from microorganisms and plants to animals and humans.

The accumulation of plastics in the soil is expected to increase due to the continued growth in the production and use of plastics, as well as the lack of effective plastic waste management strategies. A solution could be the use of biodegradable polymers, especially cellulose and its derivatives, polylactide (PLA) or poly(ε-caprolactone), the production and the use of which continue to grow. However, the breaking down of biodegradable plastics can also produce microplastic particles [267]. Given the scale of the plastics market and their degradation period, microplastic pollution is still a long-term problem.

An extensive long-term research is needed to determine the exact impact of microplastics on the environment and the health of living organisms. Only then can we be in a position to have precise impact data and determine actions leading to significant improvements in this field.

## Figures and Tables

**Table 1:** Representative PCV plasticizers [32,33].

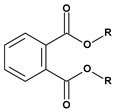	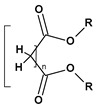		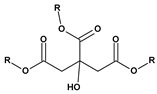	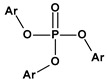
Phthalates DEHP (R = iC_8_) DIDP (R = iC_10_) DINP (R = iC_9_)	Adipates (n = 4) DINA (R = iC_9_) DIDA (R = iC_10_)	Sebacates (n = 8) DBS (R = C_4_) DOS (R = iC_10_)	Citrates TEC (R = Et)	Phosphates TCP (Ar = Tol)

**Table 2:** A summary of a water consumption and wastewater generated during the production of 1 kg of S-PVC ([80], modified).

St.	Steps of PVC Production Process	WI [kg] ^a^	WsWO [kg] ^a^
1	Ethylene (E) and Chlorine (Cl_2_) Production		
1.1.	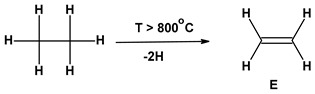	0	0
1.2	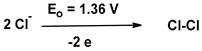	0	0
2	VCM production process	1.03	0.63
	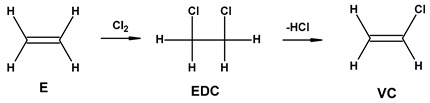		
3	PVC production process	2.24	1.83
	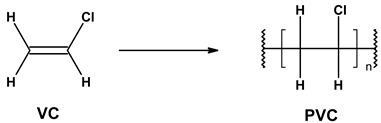		
1-3	**Total**	**2.24; 3.27**	**2.46**

**E**—**E**thylene; **EDC**—**E**thylene **D**i**C**hloride; **VC**—**V**inyl **C**hloride; **PVC**—**P**oly(**V**inyl **C**hloride); **W**I—Water Input; **Ws**W**O**—Wastewater Output. In the oxy–chlorination process, ethylene reacts with a mixture of chlorine and oxygen. ^a^ Rounded to the second decimal place. 1.2 According to [81].

**Table 3:** The assessment of PVC pipes’ environmental impact. Adapted with permission from [83]. Copyright © 2023, Elsevier.

Impact Category	Unit	Manufacturing	Use and Waste Disposal	Total Impact
Global warming	g CO_2_ eq	272,308.0	223,031.1	495,339.1
Acidification	H+ moles eq	63,922.6	25,215.8	89,138.5
Human health-cancer	g C_6_H_6_ eq	44,720.6	428.5	45,149.1
Human health-noncancer	g C_7_H_7_ eq	56,880,581.6	627,435.7	57,508,017.3
Eutrophication	g N eq	94.2	47.4	141.7
Ecotoxicity	g 2,4-D eq	3304.5	223.5	3528.0
Smog	g NOx eq	858.7	214.2	1072.9
Habitat alteration	T&E count	1.55 × 10^−13^	4.65 × 10^−13^	6.2 × 10^−13^
Ozone depletion	g CFC-11 eq	0.0008	0.004	0.00

**Table 4:** Representative occurrence of PVC in environmental waters, sediments, and organisms of marine animals.

Waters & Sediments			
Water supply		Changsha (Hunan, https://pl.wikipedia.org/wiki/Hunan, accessed on 4 October 2023), China	Yin, 2019 [101], Shen, 2021 [102]
		NW Germany	Mintenig, 2019 [103]
Surface fresh water		Wei River, Yellow River's tributary, China	Ding, 2019 [104]
		Pearl River catchment, China	Fan, 2019 [105], Yan, 2019 [106]
		Honghu Lake, China	Xiong, 2021 [107]
Sediments & surface fresh water		West Lakes, China	Jiang, 2018; [108] Wang, 2018 [109]
Surface and sub-surface seawater		Korean coastal regions	Chae, 2015; [110] Song, 2015 [111]
		Marmara Sea	Tunçer, 2018 [112]
		Kuantan of Malaysia	Khalik, 2018 [113]
		Greenland	Morgana, 2019 [114]
		Arctic Ocean	Lusher, 2015 [115]
		NW Pacific	Pan, 2019 [98]
Water column		NW Mediterranean Sea	Lefebvre, 2019 [116]
		Bohai Sea-Yellow Sea	Dai, 2018 [117]
Floating and bottom sediment microplastics		Adriatic Sea	Zeri, 2018; [118] Palatinus, 2019 [119]
		W. Mediterranean Sea	de Haan, 2019 [120]
		Cilacap, Java (Indonesia)	Syakti, 2017 [121]
Surface seawater and sediment		Melbourne coastal metropolis	Su, 2020 [122]
		Suva coastal area of Fiji	Ferreira, 2020 [123]
Bottom sediments		Arctic Ocean	Kanhai, 2019 [124]
		Norwegian fjords	Gomiero, 2019 [125]
		Venetian islands	Vianello, 2013 [126]
		Singapore coastline mangrove ecosystems	Nor, 2014 [127]
Sand seashore		Atlantic seashore, Cape Town, South Africa	Vilakati, 2020 [128]
		Atlantic seashore, Punta del Este, Uruguay	Lozoya, 2016 [129]
Marine Animals and Organisms			
Fishes	*Siganus fuscescens*	Coastal sediments, Negros, Philippine	Bucol, 2020 [130]
	*Nephrops norvegicus*	Coasts of Ireland.	Hara, 2020 [131]
	Sardines	NW Mediterranean Sea	Lefebvre, 2019 [116]
	* Triglops nybelini *, *Boreogadus saida*	Arctic Ocean	Lusher, 2015 [115]
	Various species	Australian markets	Wootton, 2021 [132]
		Suva coastal area, Fiji	Ferreira, 2020 [123]
		Markets in Fujian, China	Fang, 2019 [133]
Shellfishes	*Mytilus edulis*	Mussel and oyster farming zone, Pen-Bé, France	Phuong, 2018 [134]
	*Meretrix meretrix*	Markets in Fujian & Xiamen, China	Fang, 2019 [133]
	Various species	Sal Estuary River, Goa, India	Saha, 2021 [135]

**Table 5:** Impact of PVC microplastics (PVC-MPs) on aquatic organisms.

Organism	Genus/Species	PVC-MPs Conc. [Unit]	Effect of PVC-MPs on the Organism	Refs.
Algae	*Chlamydomonas reinhardtii*	10–200 [mg/L]	Growth inhibition; reduction in chlorophyll-A level	Wang, 2020 [161].
	*Skeletonema costatum*	1–50 [mg/L]	Inhibition of growth; inhibition of photosynthesis efficiency via decrease in chlorophyll content; adsorption, aggregation, and toxic effects on algal cells	Zhang, 2017s [163].
Corals	*Zoanthus sociatus*	10 mg/L	Increase in adhesion to coral epidermis; OS induction; changes in photosynthethic efficiency	Rocha, 2020 [164].
Plants	*Utricularia aurea*	50 [mg/L]	Growth, length, and biomass inhibition; negative effects on physiological parameters (chlorophyll content)	Zhou, 2020 [165].
Mussels	*Perna viridis*	21.6–2160 [mg/L]	Decrease in clearance, respiration rates, and byssus production; decrease in median survival times with increasing pollution by PVC	Rist, 2016 [166].
	*Mytilus galloprovincialis*	-	Accumulation in the organism	Gomiero, 2019 [167].
Arthro-pods	*Daphnia magna*	50 [mg/L]	Induction of mortality; increase in immobilization	Renzi, 2019 [168].
Fish	*Clarias gariepinus*	0.50; 1.50; 3.0 [% of diet]	Reduction of mean cell volume/cell hemoglobin values; decrease in neutrophil counts; GPx alternation (brain, gill); SOD inhibition (brain, gill); CAD reduction (brain); increase in lipid peroxidation levels (brain); AChE inhibition (brain, gill); OS induction	Iheanacho & Odo, 2020 [169].
	*Cyprinus carpio*	10–30 [% of diet]	Growth inhibition; alternation of the antioxidant activities—inverse relationship between SOD, CAT after exposition on PVC; increase in GPx activities; reduction in MDA levels; alternation of antioxidant-related gene expression in the livers of larvae; changes in transcription; vacuolation of cytoplasm in the liver under exposure over 20% additives of PVC to diet	Xia, 2020 [170].
	*Dicentrarchus labrax*	100; 500 [mg/kg diet]	Increase in the phagocytic and respiratory burst activities of head kidney leucocytes; decrease in immunity and OS induction	Espinosa, 2019 [171].
	*Dicentrarchus labrax*	0.1 [% of diet]	Histopathological changes in the ingestine	Peda, 2016 [172].
	*Etroplus suratensis*	1.0–10.8 [mg/L]	Influence on SOD activity (increase at 1.03–1.8 mg/L; decrease at 3.0–10.8 mg/L); behavioral changes (fin flickering, burst swimming, and jerking movement); decrease in red and white blood cells; changes in antioxidant enzymes	Vijayaraghavan, 2022, [151].
	*Sparus aurata*	100; 500 [mg/kg diet]	Gene expression changes: *PRDX5* (decrease); *PRDX1, PRDX3* (increase); UCP1 (up-regulation).	Espinosa, 2017 [173].

AchE—acetylcholinesterase; CAT—catalase; GPx—glutathione peroxidase; MDA—malondialdehyde; OS—oxidative stress; SOD—superoxide dismutase.

**Table 6:** Examples of methods for PVC and other plastics’ neutralization and disposal from the environment.

Method	Type	Mechanism	Refs.
chemical dechlorination	chemical neutralization	modification consisting in replacing some chlorine atoms with various nucleophilic reagents	Lu, 2019 [210]
hydrothermal dechlorination	physico-chemical neutralization	conducting modifications in supercritical or subcritical water which works as a solvent and reagent for reactions of organic compounds	Li, 2017 [211]
photodegradation	physical degradation	breaking down the chemical bonds in a polymer by ultraviolet (UV) radiation	Yousif, 2015 [212]
mechanical recycling	mechanical modification	recycling technique consisting in extruding and mixing the material with primary polymers	Sadat-Shojai, 2011 [213]
pyrolysis	physico-chemical degradation	polymer decomposition under high temperature	Yu, 2016 [21]
biodegradation	biological degradation	polymer decomposition by microorganisms such as bacteria and filamentous fungi, and organisms such as insects	Vivi et al. 2019 [214] Giacomucci, 2019 [215] Tsochatzis, 2021 [216]
biofragmentation	biological modification	the breakdown of polymers into monomers, dimers, or oligomers during a lytic process, involving decrease in the molecular weight of the polymer and oxidation of the lower-weight molecules using specific enzymes (oxidoreductases and hydrolases), as well as free radicals	Restrepo-Flórez, 2014 [217]

## Data Availability

The data are included in the text.
